# Urban Form and Environmental Characteristics as Drivers of Air Pollution Exposure Variability and Inequality in Fujian Province, China

**DOI:** 10.1029/2025GH001393

**Published:** 2025-07-17

**Authors:** Chaohao Ling, Yiqi Zhang, Qian Shen, Ruohan Dai, Bangru Lou, Yiling Kang, Shaofu He

**Affiliations:** ^1^ School of History and Geography Minnan Normal University Zhangzhou China

**Keywords:** air pollution exposure, spatial inequality, urbanization, environmental changes, network analysis

## Abstract

Air pollution, particularly fine particulate matter, poses significant health and environmental risks, with exposure levels exhibiting considerable spatial inequality. However, few studies have comprehensively examined how urban form and environmental factors influence air pollution exposure and its spatial inequality. This study investigates how urban and environmental factors affect particulate matter pollution (PM_1_, PM_2.5_, and PM_10_) and its spatial inequality across 85 counties in Fujian, China. Twelve indicators across urban form, socio‐economic, and environmental domains were analyzed using principal component analysis and partial correlation networks. Our results show that while overall air pollution levels exhibit substantial variability, spatial inequality in exposure does not always correlate directly with these levels. Notably, while urbanized counties display higher pollution exposure, significant disparities in pollution distribution are observed within regions of similar pollution levels. Urban and socio‐economic features such as population density and road density are strongly correlated with higher pollution exposure, especially in more urbanized areas. In contrast, environmental factors, such as vegetation coverage and precipitation, significantly mitigate pollution levels. Principal component analysis reveals that development density and environmental changes primarily drive overall pollution levels, while economic activity and segregation contribute to the spatial inequality of exposure. Network analysis further corroborates that high‐density urban development exacerbates pollution exposure, while socio‐economic segregation contributes to uneven distribution across the population. Our findings underscore the need for integrated urban planning strategies that address these urban and environmental factors to reduce air pollution inequality and promote more equitable urban environments.

## Key Points

- The interaction between urbanization and environmental change has led to an uneven distribution of air pollution in Fujian
- Development density and economic activity, social segregation are major drivers of inequality in air pollution exposure
- Combined environmental protection and urban planning strategies are needed to address the unequal distribution of air pollution

## 1 Introduction

Air pollution, particularly fine particulate matter—including particulate matter with an aerodynamic diameter of ≤1 μm (PM_1_), ≤2.5 μm (PM_2.5_), and ≤10 μm (PM_10_), poses significant threats to human health and the environment (Lelieveld et al., 2015). These small particles can deeply penetrate the respiratory system, reaching the lungs and spreading throughout the body via the bloodstream (Zhu et al., 2021). Long‐term exposure to these pollutants is strongly linked to numerous health issues, including cardiovascular disease, lung cancer, and respiratory disorders (Hoek et al., 2013). According to the World Health Organization, air pollution is responsible for millions of premature deaths annually, especially in low‐ and middle‐income countries, which exacerbates the burden on public health systems (Manisalidis et al., 2020). In addition to its human health impacts, air pollution also harms ecosystems, contributing to a decline in water quality, stunted plant growth, and loss of biodiversity—threatening the delicate balance of ecological systems and the sustainability of human life (Lovett et al., 2009).

While air pollution is often studied in relation to natural factors, such as hydrology, land topography, and meteorological conditions, the role of urban form and structure in shaping the spatial distribution of pollution levels is increasingly recognized (Liladhar Rane et al., 2023; Tian & Yao, 2022). Urban form refers to the spatial configuration and organization of cities, encompassing aspects like development patterns, the distribution of facilities, and economic activities (Oliveira, 2016). These urban characteristics—population distribution, development density, transportation infrastructure, and centralization of amenities—play a crucial role in influencing the spatial distribution of air pollution (Rodríguez et al., 2016). Notably, urban features such as the density of development, the public transportation network, the layout of industrial zones, and the availability and distribution of green spaces directly affect how pollution is distributed across a city (Fan et al., 2017). Generally, environmental factors such as climate conditions (temperature, precipitation) and urban greening (vegetation coverage) can significantly influence air pollution dispersion (Ortega‐Rosas et al., 2020). Increasing vegetation cover has been shown to help reduce airborne particulate matter, improving air quality (Diener & Mudu, 2021). However, climatic factors like rising temperatures and varying precipitation patterns can also affect the movement and deposition of pollutants, further complicating the issue.

The interaction between urbanization and environmental changes may interactively contribute to the spatial inequality of air pollution. Spatial inequality in air pollution refers to the unequal distribution of pollution levels across different regions within a city (Wei, 2015). Research has demonstrated that pollution is often concentrated in lower‐income, densely populated neighborhoods, while wealthier, greener areas tend to experience better air quality (Marshall et al., 2009). This disparity is partly shaped by the specific patterns of urbanization, such as high‐density development, the distribution of transportation networks, and industrial activity (Talkhabi et al., 2024). Recent studies have leveraged machine learning and high‐resolution mobility data to quantify micro‐scale exposure inequalities, revealing how urban density and socio‐economic segregation interact to shape uneven pollution distribution (Zhang et al., 2022). For instance, a study in the Bronx combined granular mobility data from over 500,000 unique users with high‐resolution air pollution measurements to assess disparities in PM_2.5_ exposure (Testi et al., 2024). The findings indicated that individuals from Hispanic‐majority and low‐income neighborhoods experienced significantly higher levels of exposure compared to other demographic groups. However, an important unresolved question is how urban characteristics—such as development density, economic activity, centrality, and segregation—combined with environmental factors, contribute to variations in pollution distribution and spatial inequalities within cities.

To address this research gap, this study investigates the extent to which urban form, socio‐economic and environmental factors, explain spatial inequality in air pollution distribution across counties in Fujian Province, China. The primary research questions guiding this study are: (a) What is the extent of spatial inequality in air pollution across counties in Fujian Province? (b) To what extent can the distribution and spatial inequality of air pollution be explained by different urban form, socio‐economic and environmental factors? To answer these questions, we use comprehensive data sets related to air pollution, including PM_1_, PM_2.5_, and PM_10_ concentrations, from the ChinaHighAirPollutants database to quantify the spatial inequality of air pollution across 85 counties in Fujian Province. In addition, we incorporate high‐resolution data on urban mobility and amenities to characterize each county's urban morphology and structure. We then explore how urban form and socio‐economic may be shaping the distribution and spatial inequality of air pollution by examining six distinct urban form and socio‐economic features (i.e., road density, conversion pressure index (CPI), point of interest (POI) density, urban centrality index (UCI), population density, and 2020 GDP) and six environmental features (mean annual temperature (MAT), precipitation (MAP), NDVI (Normalized Difference Vegetation Index), conversion pressure index (CPI), ventilation coefficient (VC) and surface air pressure(SAP)) to assess their potential relationships. We use the spatial Gini index (SGI) to quantify the spatial inequality of air pollution. We then perform principal component analysis (PCA) on the urban form and structure variables, identifying two main components: development density, environmental changes, and economic activity and segregation. Furthermore, we use network analysis to explore how the distribution of pollutants interacts with the identified PCA components, offering a more nuanced understanding of the factors driving air pollution inequality. Finally, we discuss integrated urban design strategies for addressing spatial inequality in air pollution, particularly in rapid urbanization and environmental changes.

## 2 Materials and Methods

### 2.1 Definition and Data for Air Pollution

The PM_2.5_, PM_10_, and PM_1_ data used in this study are derived from the ChinaHighAirPollutants (CHAP) data set (Wei et al., 2019; Wei, Li, Lyapustin, et al., 2021; Wei, Li, Xue, et al., 2021; Zhao et al., 2021), which provides China with high‐resolution, seamless, and long‐term ground‐level air pollution data. This data set utilizes artificial intelligence techniques to fill spatial gaps in products such as MODIS, MAIAC, and AOD by integrating data sources such as ground‐based measurements, satellite remote sensing products, atmospheric reanalysis, and emission inventories. The CHAP data set spans from 2000 to the present and covers the entire Chinese territory with a spatial resolution of 1 km. The PM_2.5_ data set has a cross‐validation coefficient of determination (*R* ^2^_cv_) of 0.92 and a root mean square error (RMSE) of 10.76 μg/m^3^, the PM_10_ data set shows an *R* ^2^_cv_ of 0.90 and an RMSE of 21.12 μg/m^3^, and the PM_1_ data set has an *R* ^2^_cv_ of 0.83 and an RMSE of 9.50 μg/m^3^. In this study, we used the annual average concentrations for 2020.

The Air Pollution Exposure (APE) is calculated using a population‐weighted exposure framework that incorporates both the pollutant concentrations and population density within a given region (Chen et al., 2022; Wu et al., 2023). The formula for APE is as follows:

(1) $$\text{APE}=\frac{\sum_{i=1}^{M}P_{i}\times C_{i}^{d}}{\sum_{i=1}^{M}P_{i}},$$

where *P*_*i*_ denotes the population of pixel *i*, $C_{i}^{d}$ denotes the concentration of the pollutant (PM_1_, PM_2.5_, or PM_10_) at pixel *i*, adjusted by a distance decay factor *d* that accounts for the influence of nearby pollution sources within a specific buffer distance (1 km is used in this study), *M* is the total number of pixels within the county, and APE is the population‐weighted air pollution exposure at a county level.

Spatial inequality of air pollution was assessed using the SGI, a widely used measure of spatial inequality that quantifies the distribution of pollution across different regions (Sousa & Nicosia, 2022). SGI is calculated by comparing the actual distribution of air pollution with a hypothetical equal distribution (Coleman et al., 2023). The formula for SGI is:

(2) $$\text{SGI}=\frac{\sum_{i=1}^{N}\sum_{j=1}^{N}w_{ij}\left|x_{i}-x_{j}\right|}{2N^{2}\overset{═}{x}},$$

where *N* is the number of spatial units (e.g., counties), $x_{i}$ is the air pollution level of area *i*, and $w_{ij}$ denotes the spatial weights derived from the adjacency matrix A, which indicates whether two areas are adjacent (binary: 1 if adjacent, 0 if not). The SGI quantifies the degree of inequality in the distribution of air pollution in each county, with values closer to 0 indicating a more equal distribution, and values closer to 1 indicating higher spatial inequality (Rey & Smith, 2013).

In this study, we use the sum of PM_1_, PM_2.5_, and PM_10_ concentrations as a composite indicator to represent overall air pollution. Each of these particulate matter fractions has distinct sources, environmental behaviors, and health implications (Chen & Lippmann, 2009; L. Wang et al., 2015). PM_1_, with its small size, can deeply penetrate the human body and contains high levels of toxic substances. PM_2.5_ is closely associated with industrial and vehicle ‐ related emissions and has long ‐ range transport potential. PM_10_ has a wide range of sources including industrial activities and road dust. By combining these three fractions, we aimed to capture the cumulative impact of different ‐ sized particulate matter on air quality in Fujian Province. This approach is consistent with previous studies that have recognized the importance of considering multiple particulate matter components for a comprehensive assessment of air pollution (Cheriyan et al., 2020; Kwon et al., 2015; D. Li et al., 2024). In addition, this approach is chosen also because the APE and SGI for each pollutant show high correlations (Figure S1 in Supporting Information S1). By summing the concentrations of PM_1_, PM_2.5_, and PM_10_, we create an index highly correlated with the APE and SGI for these pollutants, making it a robust and representative indicator for studying air pollution in the region. In the subsequent sections of the article, both the APE and SGI represent the sum of PM_1_, PM_2.5_, and PM_10_ concentrations.

### 2.2 Definition and Data for Socio‐Economic Features

*GDP*. To estimate the economic development status of each county, we used the 2020 gross domestic product (GDP) data sourced from the annual yearbook data of the respective counties.

*Population density*. To estimate population density, we used the total population data from the 2020 census data for each county. The population density at the county level was calculated by dividing the total population of the county by its land area. The land area data for each county was obtained from the respective annual yearbook data.

*Income segregation*. Income segregation refers to the spatial separation of different income groups within a geographic area (Li et al., 2022). This phenomenon occurs when people with similar income levels tend to live in distinct neighborhoods or areas, leading to the formation of economically homogeneous regions (Moro et al., 2021). In this study, we use the dissimilarity index (DI) to assess income segregation. The DI is a measure of spatial separation, indicating the degree to which two groups are distributed unevenly across different areas, with values ranging from 0 (indicating complete uniformity) to 1 (indicating complete segregation) (Kodros et al., 2022). We calculate the DI based on the proportion of low‐income populations in census tracts relative to the county level to quantify income segregation. The formula for calculating the DI is as follows:

(3) $$\text{DI}=\frac{1}{2}\sum_{i=1}^{N}\left|\frac{P_{i}}{P}-\frac{Q_{i}}{Q}\right|,$$

where $P_{i}$ denotes the number of low‐income individuals in a smaller geographic unit (such as a census tract), and P is the total number of low‐income individuals in the larger geographic unit (such as a county); $Q_{i}$ denotes the total population in the smaller geographic unit, and *Q* is the total population in the larger geographic unit. For the income data, we extracted median income information from the 2020 China Economic Yearbook. In this study, low‐income individuals are defined as those whose income is below the median income level for the respective region (Jbaily et al., 2022).

### 2.3 Definition and Data for Urban Form Features

*POI density*. To capture the distribution of physical facilities, we used open‐source data from the GAODE Web API platform (Yang et al., 2024). This data set includes comprehensive information on POIs, such as POI ID, name, geographic coordinates, address, and classification based on industry codes. The POIs in this study are categorized into basic types that are closely related to daily human activities. These types include restaurants, schools, grocery stores, churches, gas stations, pharmacies, banks, hospitals, parks, and shopping centers. We calculated the total number of POIs in each county and computed their density by dividing the number of POIs by the county's area.

*Road density*. To capture the distribution of the road network, we extracted data from OpenStreetMap for counties in Fujian Province (Liu et al., 2020). By assembling road segments, we estimated the overall road network within each county. Since the length of the road segments is uniform across the data set, we calculated the road density by dividing the total number of road segments by the area of each county.

*Urban centrality index*. We adopted the Urban Centrality Index (UCI) to describe the concentration of facilities within each county. The UCI is calculated as the product of the local coefficient and the proximity index (Pereira et al., 2013).

The local coefficient (LC) is defined as:

(4) $$\text{LC}=\frac{1}{2}\sum_{i=1}^{N}\left(k_{i}-\frac{1}{N}\right),$$

where $k_{i}$ is the number of POIs in census tract *i*, *n* is the total number of census tracts within the county. The LC measures unevenness in the distribution of POIs across different census tracts, which captures how concentrated the POIs are within each tract, with higher values indicating greater concentration.

The proximity index (PI) is defined as:

(5) $$\text{PI}=1-\frac{V}{V_{\max}},$$

(6) $$V=K^{\prime }\times D\times K,$$

where $K^{\prime }$ is the vector represents the number of POIs in each census tract, *D* is the distance matrix that represents the distances between census tracts, *K* is the vector of POI numbers, $V_{\max}$ denotes the maximum possible value of *V*, based on the hypothetical case where POIs are optimally distributed in a single central location.

The urban centrality index is then calculated by multiplying the local coefficient and the proximity index:

(7) UCI=LC×PI,

### 2.4 Definition and Data for Environmental Features

*Conversion pressure index*. To assess the environmental changes due to land conversion, we use the Conversion Pressure Index (CPI). The CPI is a composite metric that combines historical rates of human modification and the suitability for future land conversion, offering a spatially explicit measure of future land‐use pressure (Oakleaf et al., 2024). The CPI is designed to reflect the cumulative gradient of anthropogenic impacts on the environment rather than simply categorizing land‐cover changes. The CPI is derived by combining the projected human modification for 2030 (HM2030) and the Development Suitability Index (DSI) using a fuzzy sum operator. This method integrates both historical and projected land‐use changes, along with the suitability for future development, to generate a comprehensive measure of land conversion pressure. The resulting CPI values range from 0, indicating no land conversion pressure, to 1, indicating the highest potential for land degradation due to future human modification and development suitability.

*Mean annual temperature and mean annual precipitation*. The mean annual temperature (MAT) and mean annual precipitation (MAP) data for 2020 were obtained from a high‐resolution climate data set for China (Peng et al., 2019). This data set provides nationwide coverage with a spatial resolution of 1 km, significantly finer than the previously used coarser data. The accuracy of the data set was validated via cross‐validation: for MAT, the *R* ^2^_cv_ is 0.95 with a RMSE of 0.4°C; for MAP, *R* ^2^_cv_ is 0.93 with an RMSE of 15 mm.

*Normalized difference vegetation index*. The normalized difference vegetation index (NDVI) data for 2020 was sourced from a long‐term, gap‐free daily NDVI data set (H. Li et al., 2024), with a spatial resolution of 0.05°. The data set was reconstructed using a combination of valid data identification and spatiotemporal gap‐filling techniques to ensure high temporal resolution. The accuracy of the reconstructed NDVI data set was evaluated by comparing it with other established NDVI data sets. The *R* ^2^_cv_ for the reconstructed NDVI data set is 0.79, with an RMSE of 0.05.

*Ventilation coefficient*. The ventilation coefficient (VC) data, derived from the product of 10‐m wind speed and atmospheric mixed layer height, were obtained from the ERA‐Interim reanalysis data set (Balsamo et al., 2015). The VC values for each county in Fujian were calculated as the average of the four nearest grid cells based on latitude and longitude matching. The ERA‐Interim data set is widely recognized for its reliability in atmospheric dynamics research, supporting robust analysis of pollution‐dispersing capacity across the region (Jones et al., 2017).

*Surface air pressure*. The surface air pressure (SAP) data were derived from the China Meteorological Forcing Data set (CMFD) (He et al., 2020), a high‐resolution gridded product developed specifically for land process studies in China. With a spatial resolution of 0.1° and a temporal resolution of 3 hr, the CMFD integrates in situ meteorological station observations (700+ stations), satellite remote sensing (e.g., TRMM precipitation), and reanalysis data sets (e.g., GLDAS) using advanced fusion algorithms. For SAP, validation against independent station measurements across China shows a cross‐validation coefficient of determination (*R* ^2^_cv_ = 0.94) and a root mean square error (RMSE = 1.8 hPa).

### 2.5 Statistical Analysis

We employed ordinary least squares regression models to analyze the relationship between APE and SGI with various urban forms and environmental variables. The regression analysis aims to explore how urban form and socio‐economic characteristics (i.e., road density, POI density, population density) and environmental factors (i.e., MAT, MAP) influence the spatial distribution and inequality of air pollution. In the regression, we used a logarithmic transformation of values since the values of GDP, POI density, population density, MAP, SAP and VC have a much larger scale than other variables.

We employed principal component analysis (PCA) to extract latent dimensions of urban form, socio‐economic and environmental characteristics (Abdi & Williams, 2010). This approach helps reduce multicollinearity among variables such as vegetation coverage and development density. Moreover, by analyzing the principal components rather than raw indicators, we mitigate direct correlations between individual environmental variables and pollution outcomes (Figure S6 in Supporting Information S1). We also examined linear relationships between the first two principal components and APE and SGI of air pollution.

The network analysis approach provides a robust method for understanding the complex interdependencies between urban form, environmental factors, and pollution exposure. It allows us to pinpoint critical variables central to air pollution inequality, offering insights into the spatial dynamics of environmental stressors. To explore the interactions and relationships between APE and SGI of air pollution, and various influencing factors, we employed a Partial Correlation Network Analysis (Epskamp & Fried, 2018). Specifically, we used partial correlation coefficients to estimate the strength of the relationships between the key variables (such as population density, temperature, precipitation, and NDVI) and APE and SGI. We utilized a Markov random field network model to estimate the partial correlation networks, optimizing the network structure using the extended Bayesian information criterion (EBICglasso) (Zhou et al., 2024). This process involved identifying clusters of variables that responded similarly to the increasing environmental stressors, such as air pollution and urban development factors. The centrality of each variable was computed by summing the absolute values of its partial correlations with other variables, identifying the most influential attributes in determining the spatial distribution of air pollution and its inequality across regions. The network analysis quantified centrality by measuring the correlation strength with all other connected variables, with higher values signifying stronger influence.

While we recognize that methods such as geographical detectors and spatial dynamic panel models offer valuable advantages—especially in uncovering non‐linear interactions or dynamic effects—they often require finer temporal resolution or strong instrumental variables, which are not available in our data set (Fingleton, 2023). For instance, spatial dynamic panel models require repeated observations over time, which are limited in our county‐level cross‐sectional analysis (Yang et al., 2017). Together, PCA and network analysis provide a robust framework to address the study's core questions about spatial inequality mechanisms using the available data (Abson et al., 2012; Li & Fang, 2014).

All statistical analyses were performed using R version 4.3.3. Two‐sided *P*‐values <0.05 were considered statistically significant.

## 3 Results

### 3.1 Variation in Distribution and Spatial Inequality of Air Pollution Among Counties

**Figure 1:**
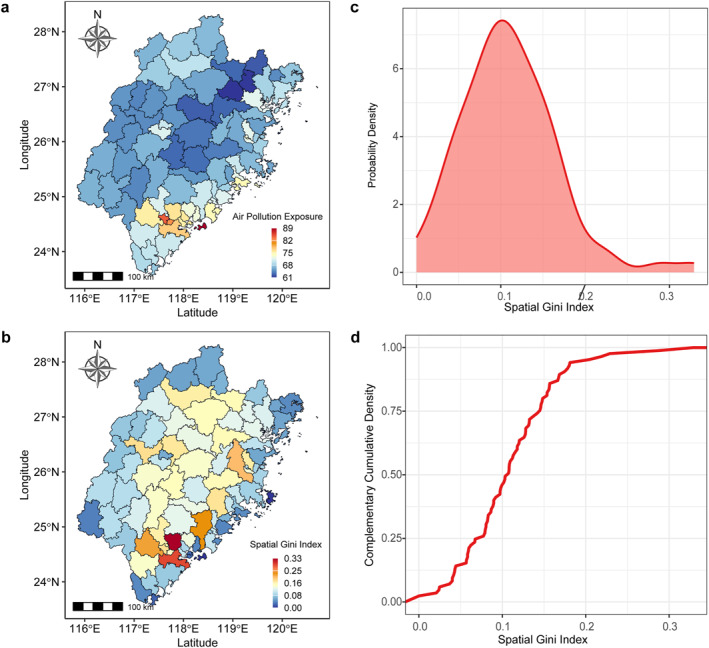
Disparities of spatial inequality in air pollution exposure among counties in Fujian Province. (a) County‐level visualization of air pollution exposure (APE). The color gradient from dark blue to dark red represents increasing levels of APE. (b) Spatial distribution of the spatial Gini index (SGI) for air pollution exposure. The color gradient from dark blue to dark red represents increasing spatial inequality in pollution exposure. (c) Probability density function of the SGI. (d) Complementary cumulative distribution function of the SGI. It should be noted that the air pollution exposure shown in the figure represents the overall pollution levels of PM_1_, PM_2.5_, and PM_10_ combined.

To investigate the variation in spatial inequality of air pollution exposure among counties in Fujian Province, the Spatial Gini Index (SGI) was calculated based on population‐weighted air pollution exposure (APE). The county‐level visualization of APE demonstrates considerable regional differences, with certain counties experiencing much higher levels of population‐weighted pollution exposure than others (Figure 1a). The mean APE for combined pollution (PM_1_, PM_2.5_, and PM_10_) is 108.52, with a standard deviation of 19.72, reflecting substantial variability in pollution exposure across counties (Table 1). Notably, the 20% of counties with the highest SGI values account for 29% of the total population‐weighted pollution exposure, whereas the 20% of counties with the lowest SGI values contribute only 11% (Figure 1b). The probability density function and complementary cumulative distribution function of the SGI reveal distinct distribution characteristics (Figure 1c). The probability density function follows a unimodal distribution, with a mean SGI of 0.15 and a standard deviation of 0.08 (Table 1). Meanwhile, the complementary cumulative distribution function exhibits a heavy‐tailed pattern, indicating that a subset of counties experiences significant spatial disparities in pollution exposure (Figure 1d).

**Table 1:** Descriptive Statistics for Air Pollution Exposure and Spatial Inequality Metrics

Exposure	Variables	Mean (SD)	Percentile				
			Min	25	50	75	Max
PM_1_	APE	21.14 (4.39)	10.79	17.82	21.94	24.41	31.73
PM_1_	SGI	0.16 (0.08)	0.01	0.1	0.15	0.21	0.63
PM_2.5_	APE	33.25 (6.19)	18.83	28.67	34.33	37.7	50.89
PM_2.5_	SGI	0.14 (0.07)	0.01	0.08	0.13	0.18	0.47
PM_10_	APE	54.13 (9.29)	30.96	47.92	55.14	60.46	77.9
PM_10_	SGI	0.16 (0.08)	0.01	0.1	0.15	0.2	0.64
Combined pollution	APE	108.52 (19.72)	60.69	94.58	111.33	122.77	157.85
Combined pollution	SGI	0.15 (0.08)	0.01	0.09	0.14	0.2	0.58

*Note.* This table presents the distribution characteristics of air pollution exposure (APE) and spatial inequality (SGI) for PM_1_, PM_2.5_, PM_10_, and combined pollution levels across counties.

The spatial distribution of SGI further reveals that high spatial inequality in pollution exposure does not necessarily correspond to the high levels of APE (Figure 1). Counties in southeastern Fujian exhibit high SGI values despite moderate APE levels, suggesting an uneven distribution of pollution burdens among the population within these areas. For instance, with southeastern coastal counties (e.g., Xiamen, Quanzhou) exhibiting both high APE (mean = 122.77) and high SGI (mean = 0.20). In contrast, some counties with high overall pollution exposure display relatively lower SGI values, indicating a more uniform distribution of pollution across the population. For instance, central to northern counties (e.g., Nanping, Sanming) show a divergent pattern: moderate to low APE (mean = 94.58) coexists with non‐negligible SGI values (0.15–0.30). Critically, the decoupling of SGI from APE is evident (Figure S5 in Supporting Information S1), and this decoupling underscores the limitations of using aggregate exposure levels alone to characterize environmental justice issues. The results for PM_1_, PM_2.5_, and PM_10_ show similar trends to the combined pollution exposure (Figures S2–S4 in Supporting Information S1, Table 1), suggesting that while the specific levels of pollutants vary, the overall patterns of spatial inequality and exposure are consistent across different particle sizes.

### 3.2 Empirical Statistics of Urban Form, Socio‐Economic and Environmental Features

**Figure 2:**
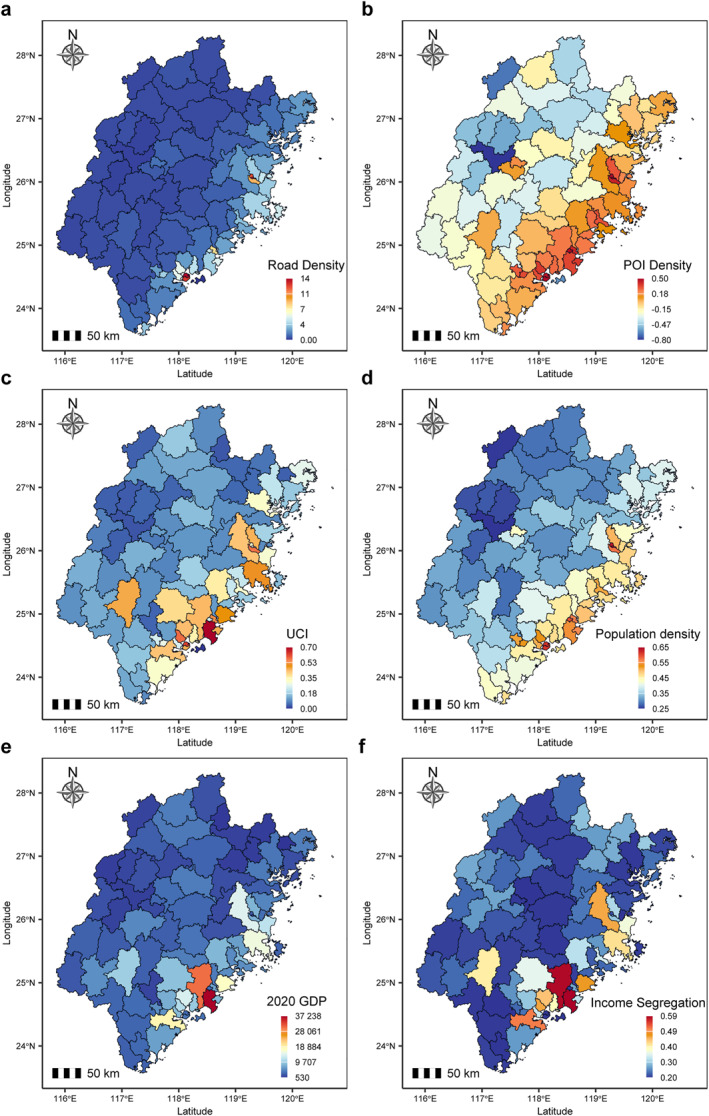
Spatial distribution of urban form and socio‐economic features among counties in Fujian Province. (a) Road density (m/km^2^). (b) POI density (points of POI/km^2^). (c) UCI. (d) Population density (100 individuals/km^2^). (e) 2020 GDP (CNY). (f) Income segregation. Due to the large‐scale differences, POI density, population density, and 2020 GDP were logarithmically transformed. See Methods for detailed metric definitions and data processing procedures. Abbreviations: POI, points of interest; UCI, urban centrality index; GDP, gross domestic product.

To examine the variations in urban form and structure across counties in Fujian Province, we mapped six key features related to urban development and socio‐economic conditions. The definition of these features and the data sets used for their development could be found in the Methods section and Table S1 in Supporting Information S1. We found the spatial distribution of these urban form and socio‐economic features showed significant heterogeneity among counties (Figure 2 and Table S2 in Supporting Information S1). Road density, POI density, population density, and 2020 GDP exhibit substantial variation, with higher values generally concentrated in coastal and urbanized regions. The urban centrality index follows a similar pattern, indicating stronger urban concentration in economically developed areas. Income segregation, however, presents a more dispersed pattern, with some counties showing notably higher levels of segregation, particularly in major metropolitan areas (e.g., counties in city of Xiamen and Quanzhou). These spatial patterns highlight the complex interactions between urbanization, economic growth, and social stratification, emphasizing that urban form and structure vary significantly across different counties.

**Figure 3:**
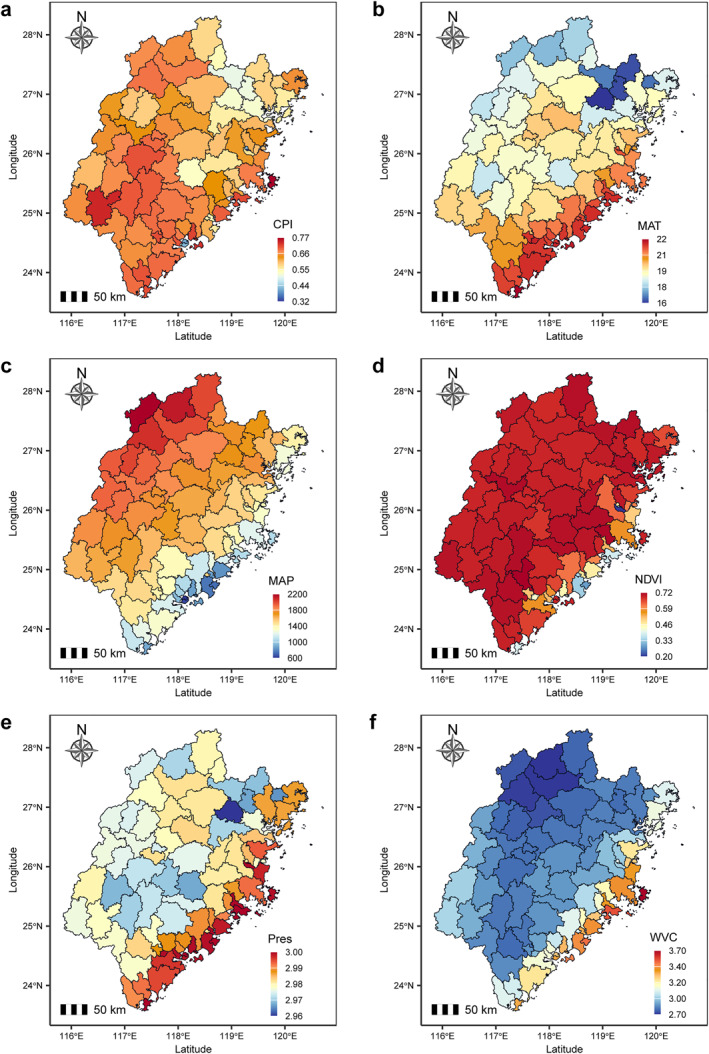
Spatial distribution of urban environmental features among counties in Fujian Province. (a) CPI. (b) MAT (°C). (c) MAP (mm). (d) NDVI. (e)SAP (hPa). (f) VC (m^2^/s). Due to the large‐scale differences, MAP, SAP and VC were logarithmically transformed. See Methods for detailed metric definitions and data processing procedures. Abbreviations: CPI, Conversion pressure index; MAT, mean annual temperature; MAP, mean annual precipitation; NDVI, normalized difference vegetation index; SAP: surface air pressure; VC: ventilation coefficient.

To further investigate the environmental characteristics shaping urban development, we analyzed four key urban environmental features across Fujian Province (Figure 3 and Table S2 in Supporting Information S1). The Conversion Pressure Index demonstrates a transparent urban‐rural gradient, with higher values concentrated in rapidly urbanizing regions, particularly in the southeastern part of the province. Mean annual temperature exhibits a latitudinal pattern, with northern counties recording cooler temperatures (mean: 16.32–21.5°C) than southern coastal areas (22.4°C maximum). Mean annual precipitation varies significantly, with southern and eastern regions receiving higher rainfall (up to 2,185.87 mm) compared to northern areas (709.16 mm minimum). NDVI values show distinct spatial variations, with lower vegetation coverage in densely populated and coastal counties (NDVI < 0.62) and higher values (0.69–0.72) in mountainous regions. Surface air pressure follows a south‐north gradient, with higher pressure in southern coastal counties and lower values in northern inland areas. Ventilation coefficient is highest in northwestern mountainous regions (VC >3.4 m^2^/s) and lowest in southeastern plains (VC <3.2 m^2^/s), reflecting enhanced atmospheric dispersion in complex terrain and reduced ventilation in urbanized lowlands.

**Figure 4:**
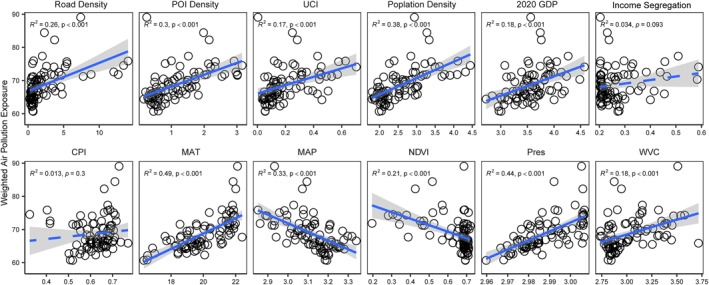
Correlation between various factors and air pollution exposure. We used the ordinary least squares (OLS) regression model to fit the data, and the confidence interval was set to 90%. Each circle represents one of the 85 counties in Fujian Province. Abbreviations: POI, points of interest; UCI, urban centrality index; GDP, gross domestic product; CPI, Conversion pressure index; MAT, mean annual temperature; MAP, mean annual precipitation; NDVI, normalized difference vegetation index; SAP: surface air pressure; VC: ventilation coefficient. It should be noted that the air pollution exposure shown in the figure represents the overall pollution levels of PM_1_, PM_2.5_, and PM_10_ combined.

**Figure 5:**
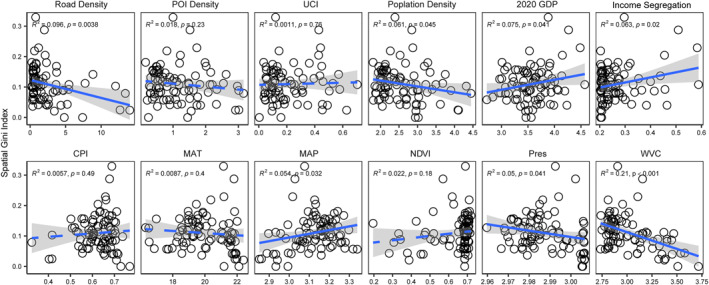
Correlation between various factors and spatial inequality in air pollution. We used the OLS regression model to fit the data, and the confidence interval was set to 90%. Each circle represents one of the 85 counties in Fujian Province. Abbreviations: POI, points of interest; UCI, urban centrality index; GDP, gross domestic product; CPI, Conversion pressure index; MAT, mean annual temperature; MAP, mean annual precipitation; NDVI, normalized difference vegetation index; SAP: surface air pressure; VC: ventilation coefficient. It should be noted that the air pollution exposure shown in the figure represents the overall pollution levels of PM_1_, PM_2.5_, and PM_10_ combined.

We further disentangled the impacts of urban form, socio‐economic, and climatic conditions on two critical dimensions of air pollution—weighted air pollution exposure (Figure 4) and spatial inequality in pollution (Figure 5)—using ordinary least squares regression. For APE, urban development intensity emerged as a dominant driver: Population density (*R* ^2^ = 0.38, *P* < 0.001), POI density (*R* ^2^ = 0.30, *P* < 0.001), road density (*R* ^2^ = 0.26, *P* < 0.001) and the urban centrality index (UCI, *R* ^2^ = 0.17, *P* < 0.001) exhibited significantly positive correlations. Climatic factors also played pivotal roles: Higher mean annual temperature (MAT, *R* ^2^ = 0.49, *P* < 0.001) and surface air pressure (SAP, *R* ^2^ = 0.44, *P* < 0.001) correlated with increased APE, likely due to reduced atmospheric dispersion, while greater mean annual precipitation (MAP, *R* ^2^ = 0.33, *P* < 0.001) and normalized difference vegetation index (NDVI, *R* ^2^ = 0.21, *P* < 0.001) mitigated exposure through wet deposition and particulate uptake. Socio‐economic variables showed weaker yet significant associations, with GDP (*R* ^2^ = 0.18, *P* < 0.001) and income segregation (*R* ^2^ = 0.034, *P* = 0.003) positively relating to APE.

In contrast, spatial inequality in pollution was shaped by a distinct set of drivers (Figure 5). The ventilation coefficient (*R* ^2^ = 0.21, *P* < 0.001) exhibited the strongest negative correlation, indicating that regions with better air dispersion capacity had more uniform pollution distribution. Socio‐economic and transportation factors played outsized roles: Road density (*R* ^2^ = 0.096, *P* = 0.004), GDP (*R* ^2^ = 0.075, *P* = 0.041), and income segregation (*R* ^2^ = 0.063, *P* = 0.02) jointly exacerbated SGI, suggesting that traffic concentration, economic clustering, and social stratification funneled pollution into disadvantaged communities. Population density (*R* ^2^ = 0.061, *P* = 0.045) and MAP (*R* ^2^ = 0.054, *P* = 0.032) showed weaker positive links, while urban form variables like POI density and UCI had negligible impacts (*P* > 0.05), highlighting that socio‐economic processes, rather than sheer development intensity, govern pollution inequality.

### 3.3 Impact of Urban Form, Socio‐Economic, and Environmental Features on Air Pollution

**Figure 6:**
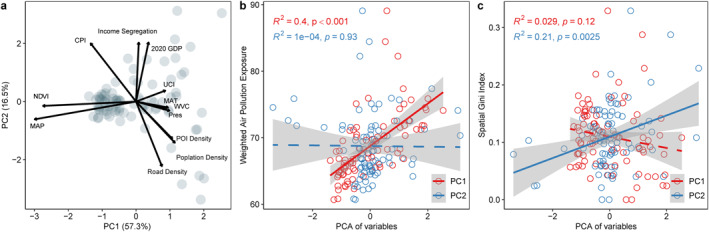
Principal component analysis and relationships with exposure and spatial inequality of air pollution. (a) Biplot of PCA results, showing the first two principal components (PC_1_ and PC_2_), which explain 57.3% and 16.5% of the variance, respectively. The direction and length of arrows indicate the contribution and influence of each variable in the PCA space. (b) Relationship between PC_1_/PC_2_ scores and combined air pollution exposure (APE, integrating PM_1_, PM_2.5_, and PM_10_). Each circle represents one of Fujian's 85 counties, with the *x*‐axis denoting the county's score on PC_1_ (red) or PC_2_ (blue) and the *y*‐axis showing its APE. OLS regression lines (solid) and 90% confidence intervals (shaded) illustrate the trend between principal components and pollution exposure. (c) Relationship between principal components and Spatial Gini Index of air pollution, with each circle representing a county and the regression analysis following the same methodology as in (b). Abbreviations: POI, points of interest; UCI, urban centrality index; GDP, gross domestic product; CPI, Conversion pressure index; MAT, mean annual temperature; MAP, mean annual precipitation; NDVI, normalized difference vegetation index; SAP: surface air pressure; VC: ventilation coefficient.

In the next step, we applied PCA to multiple urban features to identify the key factors influencing exposure and spatial inequality of combined air pollution (Figure 6). The PCA results indicate that the first principal component (PC_1_) primarily represents development density and environmental changes, while the second principal component (PC_2_) reflects economic activity and segregation. PC_1_ explains 57.3% of the variance and is mainly driven by factors such as population density, POI density, UCI, and mean annual temperature, reflecting urbanization intensity and environmental variability (Figure S7 in Supporting Information S1). PC_2_, which accounts for 16.9% of the variance, is primarily influenced by road density, CPI, income segregation, and GDP, representing economic activity and socio‐spatial segregation (Figure S7 in Supporting Information S1).

Regression analyses further revealed distinct relationships between PCA components and air pollution exposure (Figure 6). PC_1_ exhibits a significant positive correlation with APE (*R* ^2^ = 0.40, *P* < 0.001), indicating that areas with high development density and substantial environmental changes tend to experience higher pollution levels. In contrast, PC_2_ shows no significant association with APE (*R* ^2^ = 0.01, *P* = 0.93). However, when analyzing the spatial inequality of pollution, PC_2_ plays a more prominent role. The regression results show a significant positive correlation between PC_2_ and SGI (*R* ^2^ = 0.21, *P* < 0.01), indicating that higher levels of road density, CPI, economic activity, and segregation contribute to a more uneven spatial distribution of pollution. Conversely, PC_1_ has a negligible impact on SGI (*R* ^2^ = 0.03, *P* = 0.12).

**Figure 7:**
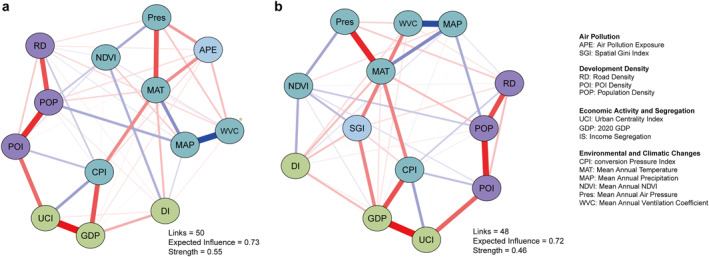
Partial correlation networks of exposure and spatial inequality of air pollution. (a) Partial correlation network for air pollution exposure (APE), nodes represent key variables. Edges indicate partial correlations, with red lines representing positive associations and blue lines representing negative associations. The thickness of the edges denotes the strength of the relationships. (b) Partial correlation network for spatial Gini index (SGI) of air pollution. It should be noted that the air pollution exposure shown in the figure represents the overall pollution levels of PM_1_, PM_2.5_, and PM_10_ combined.

Finally, we conducted network analyses of the population‐weighted exposure and spatial inequality of air pollution (Figure 7). Supporting previous results, the network analysis highlights distinct correlations between APE, SGI, and various urban morphological structures or environmental factors. The APE network (Figure 7a) consists of 50 links, with an expected influence of 0.73 and a strength of 0.56. In contrast, the SGI network (Figure 7b) contains 48 links, with an expected influence of 0.72 and a strength of 0.46. These results indicate that while both networks capture key interactions, APE exhibits slightly stronger overall connectivity.

In the APE network, variables associated with development density—such as road density (RD), point of interest density (POI), and population density (POP)—exhibit significant positive associations with APE. This dovetails with PCA results, where PC1 (urbanization intensity and environmental variability) was strongly correlated with APE. Moreover, environmental variables such as temperature (MAT), NDVI, surface air pressure (SAP), and ventilation coefficient (VC) are linked to air pollution exposure, further underscoring the role of climatic and ecological factors in shaping pollution patterns. For the SGI network, economic activity and segregation factors, including urban centrality index (UCI), GDP, and income segregation (IS), show stronger associations. This supports the PCA findings that PC_2_ (economic activity and socio‐spatial segregation) significantly influences SGI. The network structure suggests that regions with more significant economic disparity and urban centrality tend to experience more uneven pollution distribution. Bootstrap analysis (Figures S8 and S9 in Supporting Information S1) confirms the robustness of these network structures. The stability of expected influence and strength across resampled data sets indicates that key variables maintain their relative importance even under different sampling conditions. Overall, the network analysis reinforces the PCA findings, demonstrating that high‐density urban development primarily drives pollution exposure, while economic disparities and segregation contribute to the uneven spatial distribution of pollution.

## 4 Discussion

The results indicate substantial spatial inequality in air pollution exposure, particularly when examining the variation in the SGI among counties. The high SGI values in some areas demonstrate that even regions with moderate pollution levels may experience significant disparities in pollution exposure within the population. This highlights that a county's overall pollution exposure level does not necessarily correlate with the degree of spatial inequality, a finding that calls for deeper consideration of population distribution, urbanization patterns, and socio‐economic factors in shaping these disparities. Urbanization and industrialization, which are typically more concentrated in coastal and urbanized areas, play a crucial role in the levels of pollution exposure (Han et al., 2018). However, the spatial distribution of pollution exposure can be more complex than expected, as shown by the southeastern counties of Fujian, where pollution inequality is high despite moderate overall pollution levels. One prominent factor is the role of urban form and structure. As discussed in various studies, urbanization has been directly linked to increased levels of air pollution, with factors such as population density, road networks, and land use contributing to both the overall pollution levels and their distribution across cities (Liang et al., 2019; Zhang et al., 2022). In our study of Fujian Province, this aligns with the observation that more densely populated areas tend to experience higher levels of pollution exposure. This effect is magnified by urban sprawl, which complicates efforts to manage pollution as it spreads over larger areas with less efficient infrastructure (Shohan et al., 2024). Notably, polycentric urban structures—where multiple centers of activity and employment exist—are shown to exacerbate pollution in specific contexts due to the increased traffic and industrial activity concentrated around these centers (Wang et al., 2024).

However, it is essential to recognize that urban form and structure alone does not fully account for spatial inequalities in air pollution exposure. The co‐occurrence of high SGI and APE in southeastern counties highlights the compounding effects of urban densification and socio‐economic stratification. For instance, Xiamen City's high POI density and income segregation create “pollution hotspots” in low‐income neighborhoods near ports and industrial zones (Figure 2), a pattern mirrored in the network analysis linking economic activity to SGI (Figure 7b). This phenomenon also confirms the hypothesis that urbanization models need to be combined with social equity strategies in order to effectively alleviate pollution inequality (Zhang et al., 2022). The moderate SGI in northern Fujian with low air pollution exposure can be attributed to the region's unique topography, vegetation coverage, and urbanization patterns. Northern Fujian features high NDVI values (0.6–0.8), indicating dense vegetation that effectively mitigates overall APE through particulate matter absorption and enhanced air dispersion. However, despite low population density (Figure 2d), patchy urban clusters in mountainous areas—such as downtown Nanping—exhibit concentrated POI density and localized industrial activities, creating uneven pollution hotspots within otherwise clean rural landscapes. This spatial heterogeneity in pollution distribution elevates the SGI to moderate levels (0.1–0.2, Figure 1b), even as regional APE remains low. Additionally, while the region's high ventilation coefficient (Figure 3e) reduces average pollution exposure, seasonal temperature inversions in mountain valleys may trap pollutants locally, further contributing to within‐county inequality. These findings align with the network analysis, which shows vegetation strongly mitigates APE but has weaker effects on SGI, while localized economic activity drives spatial inequality (Figures 5 and 7). Notably, while NDVI correlates negatively with particulate matter exposure, this relationship may vary by vegetation type. For example, certain plant species (e.g., conifers) emit biogenic volatile organic compounds (BVOCs) that can enhance ozone formation under specific meteorological conditions (Šimpraga et al., 2019). In Fujian's context, the dominance of broadleaf forests in high‐NDVI areas (e.g., Wuyi Mountains) likely minimizes such trade‐offs, as these ecosystems prioritize particulate matter retention over BVOC emissions (Barwise & Kumar, 2020).

Our analysis of urban form and structure suggests that development density (captured through indicators such as population density, road density, and POI density) is a significant factor contributing to higher levels of air pollution exposure. The higher pollution exposure in areas with dense populations aligns with findings from other regions that show a strong correlation between urbanization and pollution levels. This is particularly relevant in the context of rapid urban growth in China and other developing regions, where economic development often exacerbates environmental stress (Udemba et al., 2024). Emissions from traffic and industrial activities are major contributors to urban air pollution, particularly in highly urbanized areas where dense populations, transportation networks, and industrial hubs converge, leading to higher exposure levels (Crippa et al., 2021). The correlation between GDP and air pollution exposure further supports this, suggesting that regions with higher levels of industrial activity and economic activity tend to have higher pollution levels. However, it is important to note that while economic activity and urban density are significant predictors of air pollution exposure, the relationship is not always linear, and factors such as land use and local governance policies also play essential roles in mitigating or exacerbating these trends (Liu et al., 2024).

The PCA identified two main drivers of air pollution exposure and spatial inequality: development density and economic activity/segregation. The findings suggest that urban areas with higher development densities and significant environmental changes are more likely to experience higher pollution exposure, while economic disparities and segregation contribute to the uneven spatial distribution of pollution. These insights emphasize the importance of considering both the physical and socio‐economic aspects of urban development when designing strategies to mitigate air pollution and its spatial inequality. The need for integrated urban design strategies that address both environmental and social dimensions of urbanization is evident, as urban form and structure cannot be separated from the social and economic processes shaping them (Ragazou et al., 2024). Furthermore, the network analysis of the population‐weighted exposure and spatial inequality of air pollution supports these findings, showing that development density and environmental factors like temperature and vegetation coverage are closely linked to air pollution exposure. The identification of economic activity and segregation as key factors influencing pollution inequality in the SGI network reinforces the idea that addressing pollution inequality requires tackling underlying socio‐economic disparities (Ganzleben & Kazmierczak, 2020).

In densely urbanized regions, environmental health disparities often intersect with racial, ethnic, and socio‐economic inequalities (Alvarez & Evans, 2021). For instance, in studies from China (Wang et al., 2025) and the United States (Collins et al., 2022), significant disparities in air pollution exposure are observed between various racial and ethnic groups, particularly affecting marginalized populations. Historical patterns of segregation, such as redlining in the U.S., have long contributed to disproportionate exposures to harmful pollutants like PM_2.5_ and NO_2_ (Lane et al., 2022). The issue of multiple environmental burdens, such as air pollution, noise, and heat stress, highlights the multifaceted nature of environmental inequality (Chen et al., 2024; Hua et al., 2024). In cities like Dortmund, Germany, studies have shown that areas with a high social vulnerability (e.g., lower socio‐economic status, immigrant populations) also face higher cumulative exposure to environmental hazards (Riedel et al., 2014). These findings align with the concept of “hotspots”—specific areas where multiple environmental stressors overlap, exacerbating health risks for the most vulnerable populations (Huggins et al., 2022). This underscores the need for holistic assessments that account for both environmental and socio‐economic factors, which can inform policies aimed at reducing these inequalities.

Interestingly, our study revealed a weaker‐than‐expected correlation between urban form and air pollution exposure inequality. While urbanization and economic activity strongly determine overall pollution levels, their role in shaping intra‐population exposure disparities is more nuanced. Economic activity influences air pollution and spatial inequality through dual pathways: 1. Direct emission enhancement via industrial, commercial, and traffic activities in high‐GDP regions, evidenced by the positive correlation between GDP and APE and PC1 (urban development density) explaining 57.3% of APE variance; 2. Indirect exacerbation of exposure inequality through socio‐spatial segregation, where economic hubs cluster industrial/transport corridors in low‐income neighborhoods (e.g., southeastern coastal counties), creating “pollution hotspots” in disadvantaged areas while wealthier regions benefit from higher vegetation coverage and ventilation (Yasin et al., 2025). SGI network analysis corroborates this, showing GDP and income segregation as central drivers of spatial disparities, highlighting that economic clustering amplifies pollution inequities via social marginalization. Thus, while urban form dictates pollution levels, socio‐economic stratification inherent in economic systems governs their uneven distribution, necessitating policies targeting both emission reduction and social equity (Hajat et al., 2015).

Our results have significant implications for urban planning and policy. As cities continue to grow, it is crucial to develop urban policies that focus on reducing spatial inequality in air pollution exposure. This involves not only improving the environmental quality of high‐density urban areas but also ensuring that vulnerable populations are not disproportionately burdened by pollution. Urban planners should consider policies that promote sustainable urban growth, such as mixed‐use development, enhanced public transportation, and green infrastructure, which can reduce pollution levels and improve overall urban livability (Rui & Othengrafen, 2023). In our analysis, it is evident that factors such as temperature, precipitation, vegetation cover, atmospheric circulation dynamics (reflected by surface air pressure characteristics), and air diffusion capacity (represented by ventilation coefficient ‐ related patterns) are key determinants of variations in pollution exposure. These elements interact to shape the complex spatial patterns of air pollution, underscoring the necessity of integrating comprehensive environmental considerations into urban planning and pollution mitigation strategies (Bai et al., 2017). For regions like Fujian Province, where significant environmental gradients exist between coastal and inland areas—encompassing not just climatic and ecological disparities but also differences in air pressure and ventilation patterns—such integration is especially vital.

Several limitations should be noted in this study. First, the absence of wind direction and wind speed data from some meteorological stations limited our ability to fully capture the transport pathways of air pollutants, although we used the ventilation coefficient as a proxy for wind speed. Second, relying solely on annual average temperature and precipitation may overlook the dynamic impacts of seasonal climate variations (e.g., monsoon patterns, temperature inversions) and extreme weather events (Zhisheng et al., 2015), which can significantly influence short‐term pollution dispersion and accumulation processes. Third, while multivariate analysis was conducted, causal inference remains challenging due to potential confounders, and spatial heterogeneity across regions may not have been fully accounted for. Future research could compare these patterns with other Chinese provinces or megacity clusters to explore broader urbanization‐pollution relationships. Fourth, the use of 2020 data, during the COVID‐19 pandemic, introduces biases as pollution levels were significantly influenced by reduced human activity. Although, we chose to use 2020 as a reference year because it allowed us to account for the exceptional impact of the pandemic on urban dynamics, which itself is a unique aspect that can inform future urban planning and pollution management strategies (Sharifi & Khavarian‐Garmsir, 2020). Future research should consider data from pre‐ and post‐pandemic periods to better capture long‐term trends in pollution exposure inequalities. Finally, we acknowledge that areas with higher vegetation coverage may also correspond to regions of intensified human activity, such as urban parks or green infrastructure in dense urban zones (J. Wang et al., 2015). While PCA and network analysis allows us to abstract complex interactions among correlated variables, potential endogeneity cannot be fully eliminated (Borsboom et al., 2021; Granato et al., 2018). Future work may incorporate instrumental variable approaches or longitudinal data to better isolate causal pathways (Burgess et al., 2015).

## 5 Conclusions

In conclusion, this study examines the relationship between urban form and structure and the spatial inequality of air pollution exposure in Fujian Province, emphasizing the differing impacts of urban form, structure, and environmental features on air pollution exposure and its spatial inequality. While urbanization and economic activity significantly contribute to higher pollution levels, the role of socio‐economic factors such as income segregation and urban centrality in shaping the unequal distribution of air pollution exposure cannot be overlooked. Our findings suggest that reducing air pollution inequality requires policies that not only aim at reducing overall pollution levels but also address the socio‐spatial disparities that exacerbate the unequal distribution of pollution exposure.

Practical strategies to achieve this include: First, integrating green infrastructure into urban planning, such as establishing connected green spaces and vegetative barriers in high‐exposure neighborhoods (Labib et al., 2021). Studies show that vegetation barriers can reduce particulate matter by 7%–24% downwind, making this approach particularly impactful in economically disadvantaged areas with limited green space (Ranasinghe et al., 2019). Second, promoting transit‐oriented development (TOD) to mitigate pollution from transportation networks. Integrating affordable housing with transit infrastructure through TOD can simultaneously address exposure inequality and mobility poverty, ensuring equitable access to clean air and transportation (Lyu et al., 2025). By prioritizing such evidence‐based strategies and accounting for environmental dynamics like ventilation patterns and atmospheric conditions, urban planners can create more targeted, inclusive solutions to reduce air pollution disparities and foster healthier, more sustainable urban environments (Nieuwenhuijsen et al., 2024). Future urban planning efforts should incorporate both environmental and socio‐economic considerations to mitigate the spatial inequalities in air pollution exposure and foster more equitable urban environments.

## Conflict of Interest

The authors declare no conflicts of interest relevant to this study.

## Supporting information

Supporting Information S1

## Data Availability

The air pollution data sets (PM_1_ (Wei & Zhanqing, 2019), PM_2.5_ (Wei & Zhanqing, 2024a), and PM_10_ (Wei & Zhanqing, 2024b)) supporting this study are sourced from the ChinaHighAirPollutants (CHAP) data set, and were used under the current study's license. Other data (GDP, population data, road density, points of interest) are publicly available and sourced from the China Yearbook (e.g., https://www.stats.gov.cn/sj/ndsj/), OpenStreetMap (https://www.openstreetmap.org/), and Gaode Map API platform (https://gaode.com/). The combined data set and R codes used for the statistical analysis in this research have been deposited on Figshare (https://doi.org/10.6084/m9.figshare.28424954.v2 (Ling et al., 2025)).
