# Supplementary material for: Urban Form and Environmental Characteristics as Drivers of Air Pollution Exposure Variability and Inequality in Fujian Province, China
Source: Geohealth. 2025 Jul 17;9(7):e2025GH001393. doi: 10.1029/2025GH001393 (PMC12268378; doi:10.1029/2025GH001393)
Supplement: Supplementary file 1 — Supporting Information S1 [file GH2-9-e2025GH001393-s001.docx]

*Geohealth*

Supporting Information for

**Urban form and environmental characteristics as drivers of air pollution exposure variability and inequality in Fujian Province**

**Chaohao Ling^1,*^, Yiqi Zhang^1^, Qian Shen1, Ruohan Dai^1^, Bangru Lou^1^, Yiling Kang^1^, Shaofu He^1,*^**

^1^ School of History and Geography, Minnan Normal University, Zhangzhou, 363000, China.

Corresponding author: Chaohao Ling (304702807@qq.com); Shaofu He (hsf1642@mnnu.edu.cn)

**Contents of this file**

Figures S1 to S9

Tables S1 to S2

**Supplementary Figures**


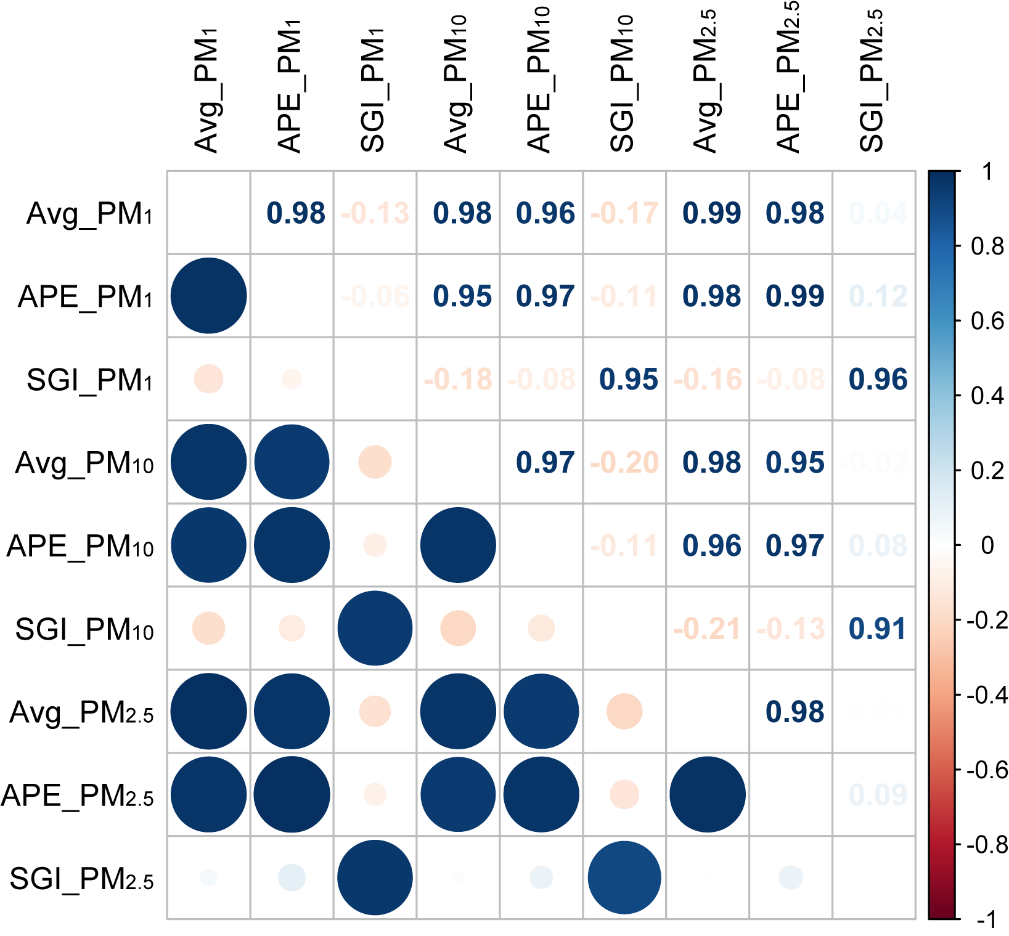


**Supplementary Figure S1. Correlation matrix of air pollution variables.** This figure displays the pairwise Spearman correlation coefficients between different air pollution variables: average concentrations (Avg), population-weighted exposure (APE), and spatial Gini index (SGI) for PM_1_, PM_10_, and PM_2.5_. The size and color of each circle represent the strength of the correlation, with dark blue indicating a strong positive correlation and light orange showing a weak or negative correlation.


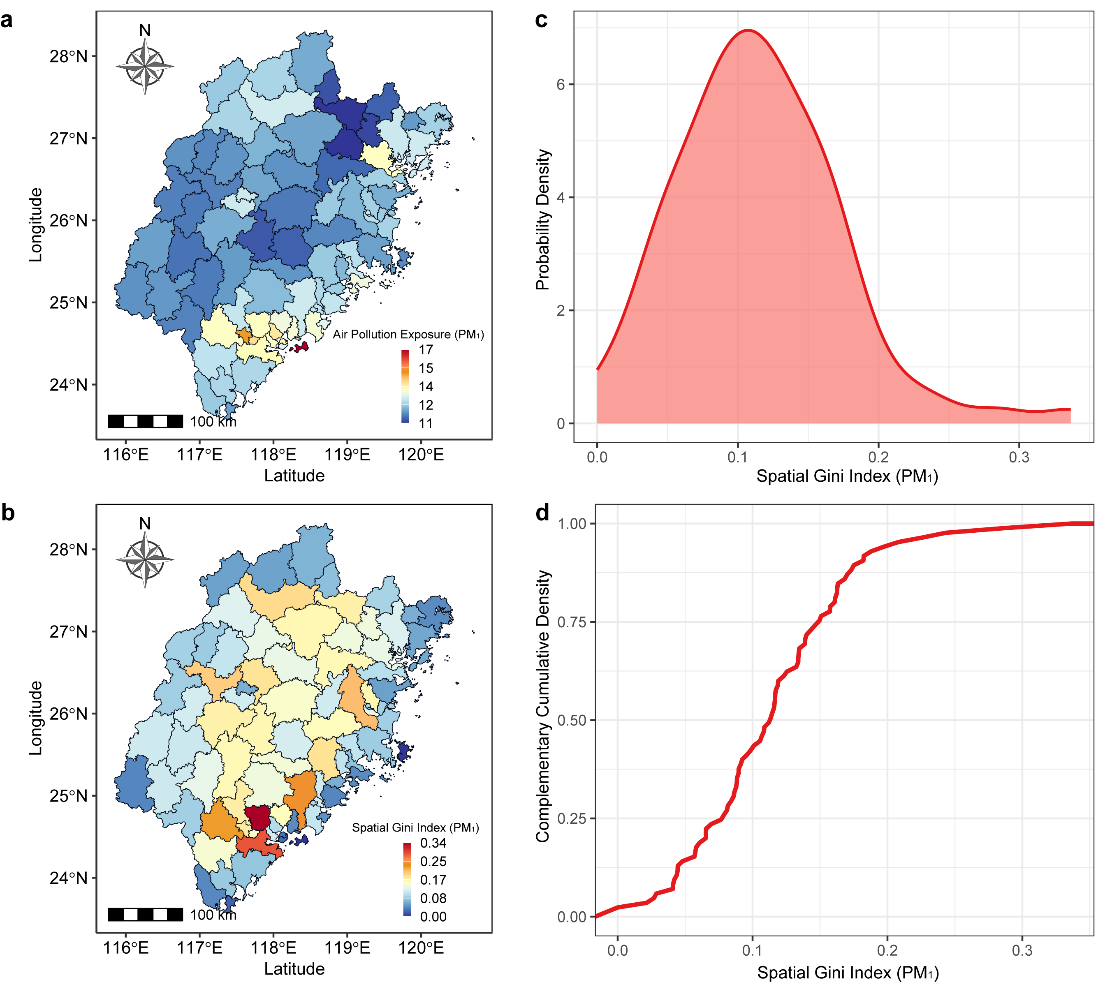


**Supplementary Figure S2. Disparities of spatial inequality in PM_1_ pollution exposure among counties in Fujian Province. (**a) County-level visualization of air pollution exposure (APE) of PM_1_. The color gradient from dark blue to dark red represents increasing levels of APE. (b) Spatial distribution of the spatial Gini index (SGI) for PM_1_ exposure. The color gradient from dark blue to dark red represents increasing spatial inequality in pollution exposure. (c) Probability density function of the SGI. (d) Complementary cumulative distribution function of the SGI.


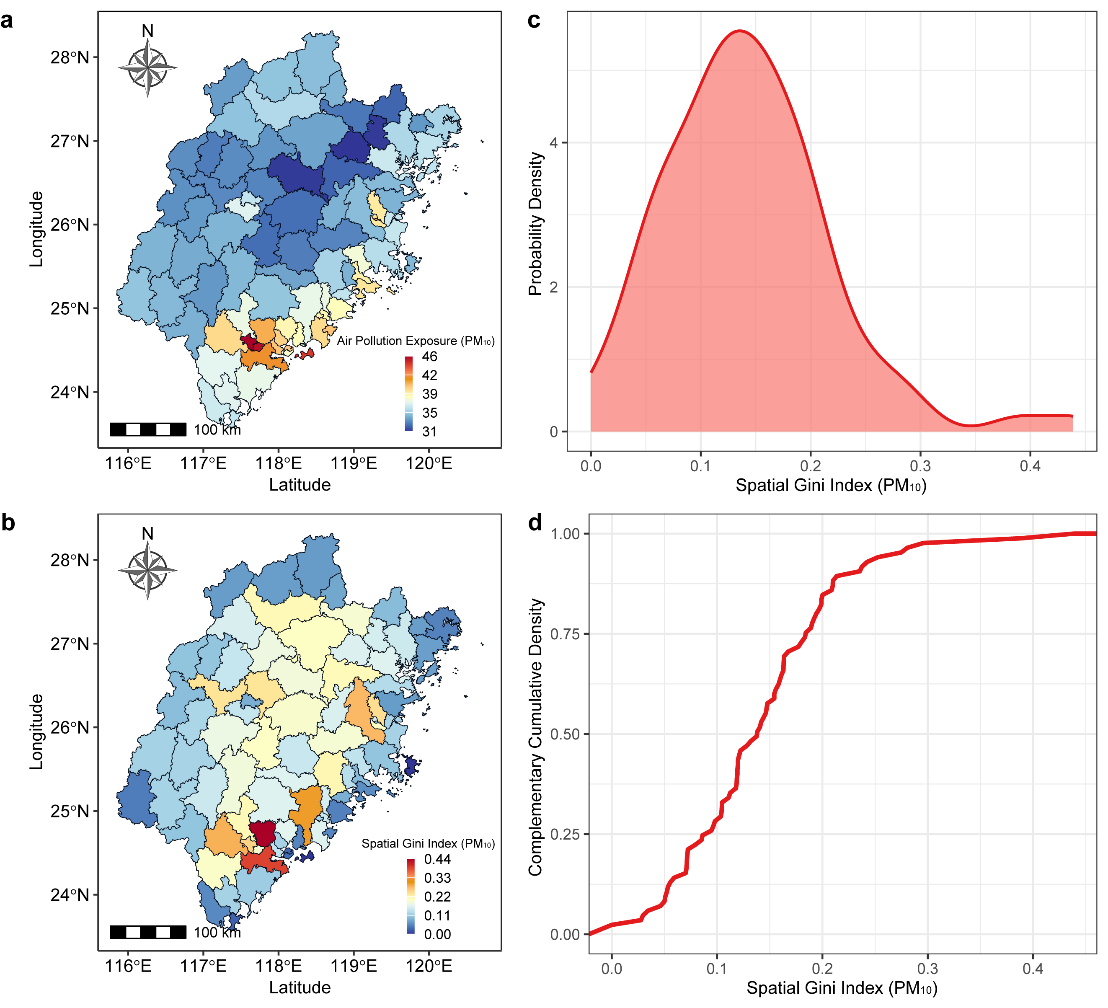


**Supplementary Figure S3. Disparities of spatial inequality in PM_10_ pollution exposure among counties in Fujian Province. (**a) County-level visualization of air pollution exposure (APE) of PM_10_. The color gradient from dark blue to dark red represents increasing levels of APE. (b) Spatial distribution of the spatial Gini index (SGI) for PM_10_ exposure. The color gradient from dark blue to dark red represents increasing spatial inequality in pollution exposure. (c) Probability density function of the SGI. (d) Complementary cumulative distribution function of the SGI.


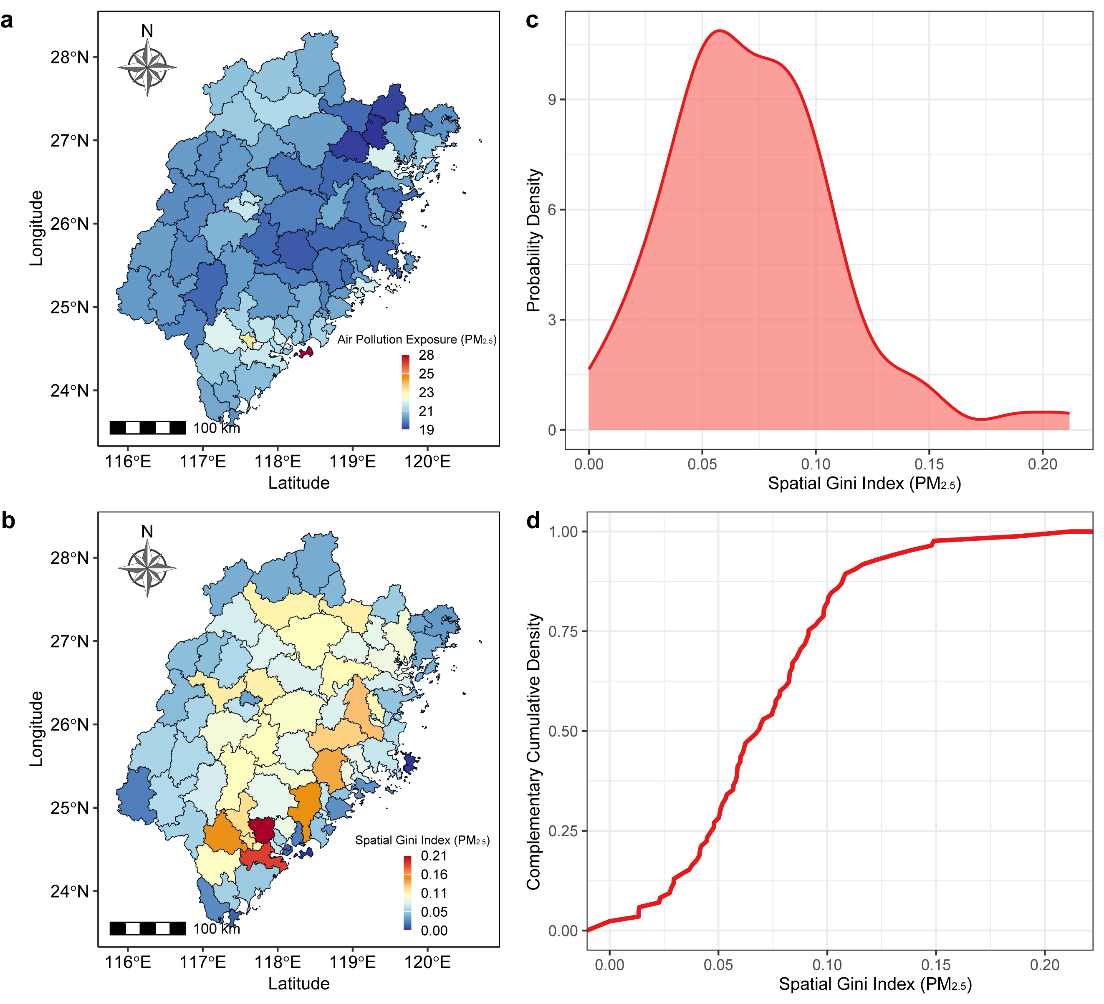


**Supplementary Figure S4. Disparities of spatial inequality in PM_2.5_ pollution exposure among counties in Fujian Province. (**a) County-level visualization of air pollution exposure (APE) of PM_2.5_. The color gradient from dark blue to dark red represents increasing levels of APE. (b) Spatial distribution of the spatial Gini index (SGI) for PM_2.5_ exposure. The color gradient from dark blue to dark red represents increasing spatial inequality in pollution exposure. (c) Probability density function of the SGI. (d) Complementary cumulative distribution function of the SGI.


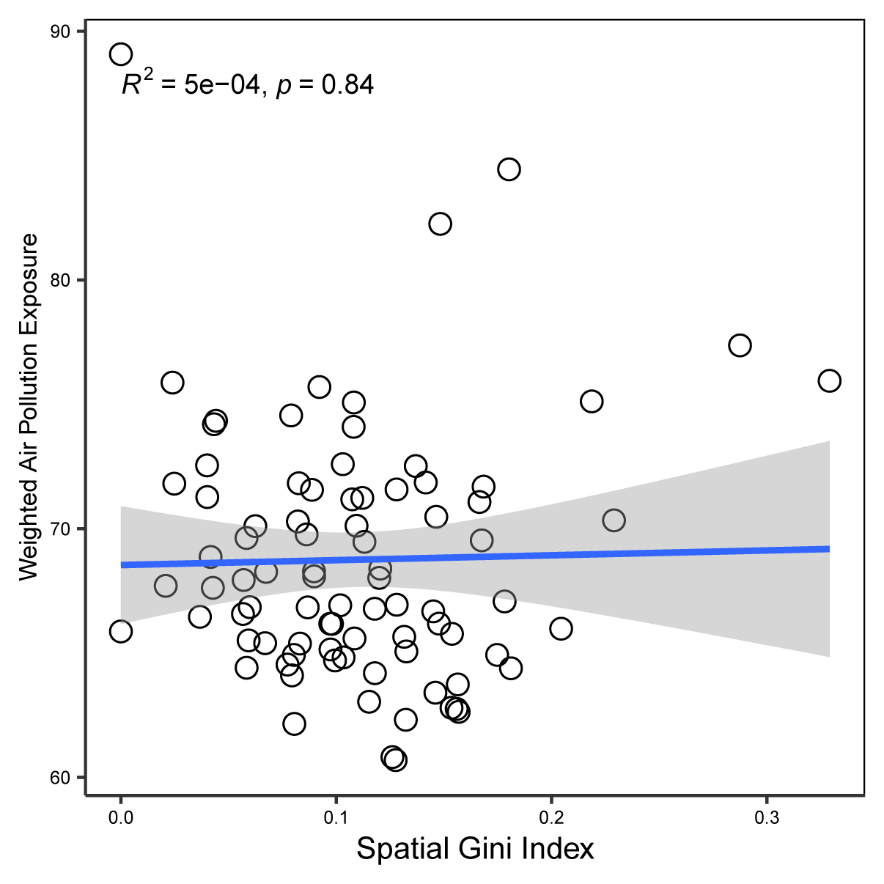


**Supplementary Figure S5. Correlations between APE and SGI of different counties across Fujian Province.**


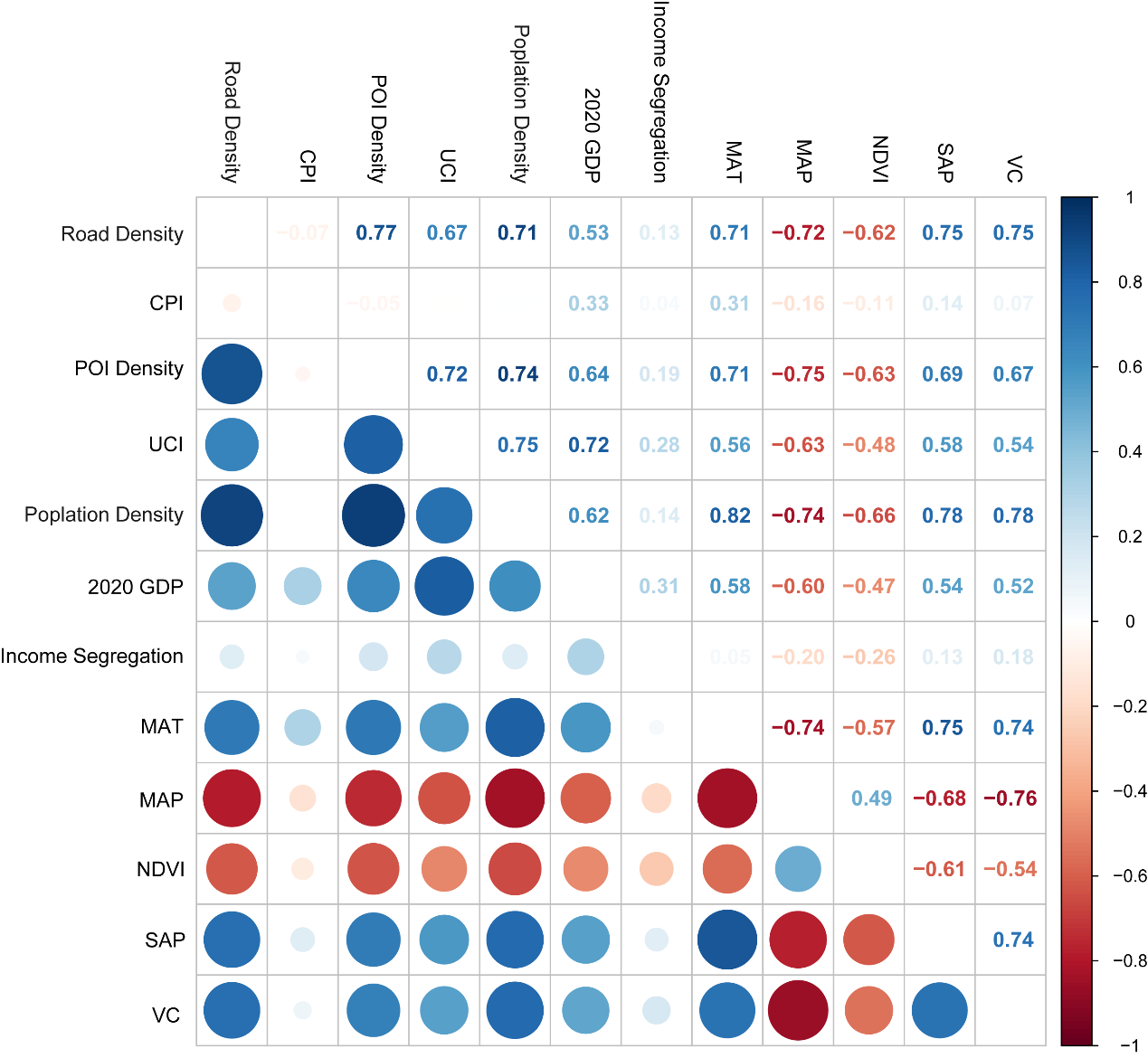


**Supplementary Figure S6. Correlation matrix of urban form, structure and environmental variables.** This figure displays the pairwise Spearman correlation coefficients between different urban features. The size and color of each circle represent the strength of the correlation, with dark blue indicating a strong positive correlation and light orange showing a weak or negative correlation.


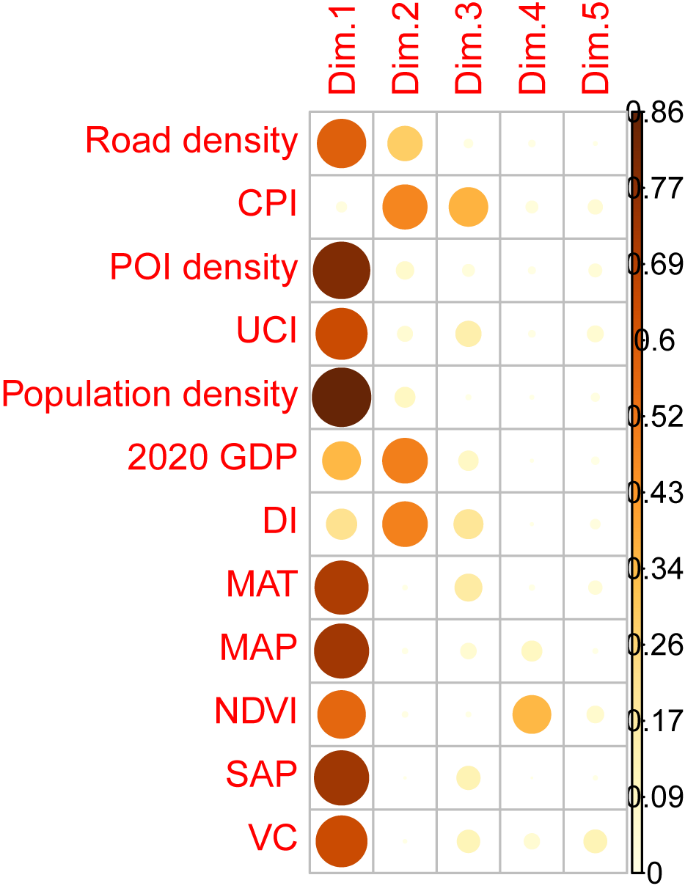


**Supplementary Figure S7. Variable loadings on principal components (PCs) from principal component analysis (PCA).** Dimensions (Dim.1–Dim.5) correspond to the first five principal components, with Dim.1 = PC1, Dim.2 = PC2, etc. Numerical values adjacent to each variable name indicate the absolute loading of the variable on the corresponding principal component (higher values signify stronger contribution to the component). Variables are sorted by loading magnitude within each dimension. Abbreviations: POI, points of interest; UCI, urban centrality index; GDP, gross domestic product; CPI, Conversion pressure index; MAT, mean annual temperature; MAP, mean annual precipitation; NDVI, normalized difference vegetation index; SAP: surface air pressure; VC: ventilation coefficient.


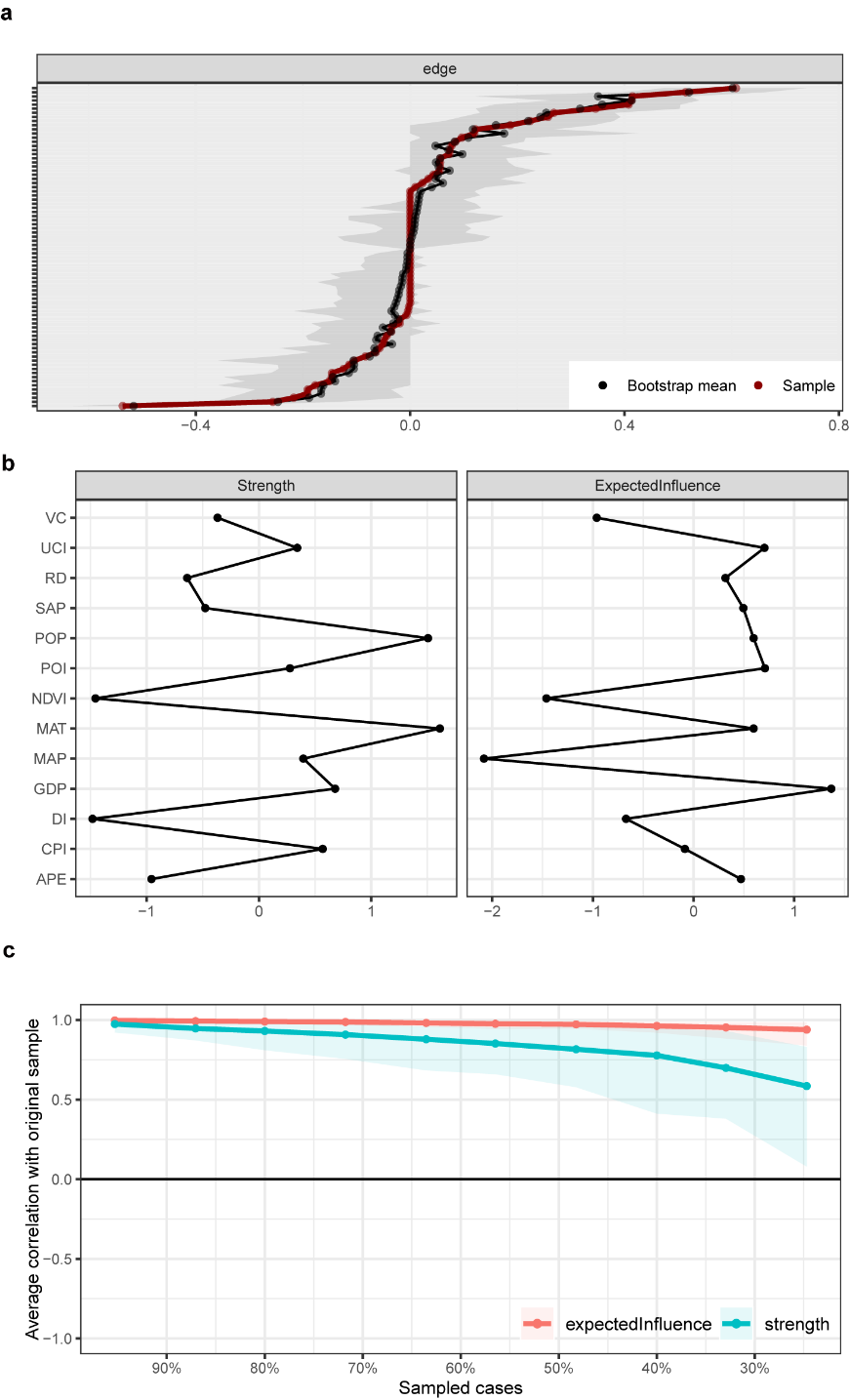


**Supplementary Figure S8. Bootstrap analysis and variable importance related to Fig 7a.** (a) Bootstrap Confidence Curve: The black dots represent the bootstrap mean for the sampled cases, while the red line shows the sample curve. The shaded area indicates the 95% confidence interval for the bootstrap results, illustrating the robustness of the model's results. (b) Strength and Expected Influence of Variables: Two plots showing the strength and expected influence of various urban form, structure, and environmental variables (UCI: Urban Centrality Index, RD: Road Density, POP: Population Density, POI: Points of Interest Density, NDVI: Normalized Difference Vegetation Index, MAT: Mean Annual Temperature, MAP: Mean Annual Precipitation, APE: Air Pollution Exposure, GDP: Gross Domestic Product, DI: Development Index, CPI: Conversion Pressure Index, SAP: surface air pressure, VC: ventilation coefficient). (c) Model Stability: The plot illustrates the average correlation between the sampled cases and the original sample as a function of the percentage of sampled cases. The lines represent expected influence (red) and strength (blue), with the shaded areas indicating the variability, showing that the model's performance remains stable across different sample sizes.


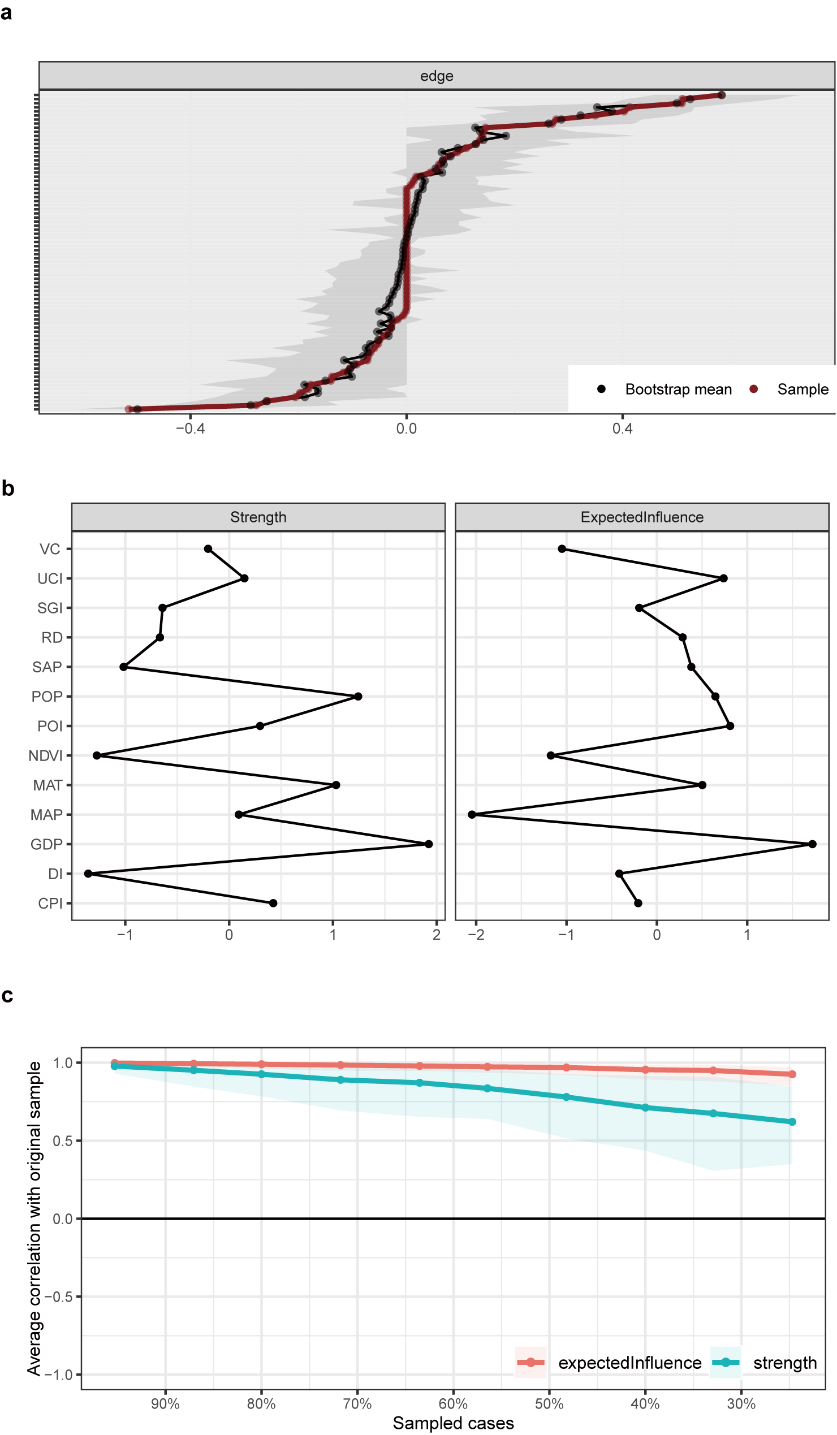


**Supplementary Figure S9. Bootstrap analysis and variable importance related to Fig 7b.** (a) Bootstrap Confidence Curve: The black dots represent the bootstrap mean for the sampled cases, while the red line shows the sample curve. The shaded area indicates the 95% confidence interval for the bootstrap results, illustrating the robustness of the model's results. (b) Strength and Expected Influence of Variables: Two plots showing the strength and expected influence of various urban form, structure, and environmental variables (UCI: Urban Centrality Index, RD: Road Density, POP: Population Density, POI: Points of Interest Density, NDVI: Normalized Difference Vegetation Index, MAT: Mean Annual Temperature, MAP: Mean Annual Precipitation, APE: Air Pollution Exposure, GDP: Gross Domestic Product, DI: Development Index, CPI: Conversion Pressure Index, SAP: surface air pressure, VC: ventilation coefficient). (c) Model Stability: The plot illustrates the average correlation between the sampled cases and the original sample as a function of the percentage of sampled cases. The lines represent expected influence (red) and strength (blue), with the shaded areas indicating the variability, showing that the model's performance remains stable across different sample sizes.

**Supplementary Tables**

**Table S1. Summary statistics for urban form, socioeconomic and environmental features**. This table offers data resolution and reference point for the literature sources that informed our feature screening.

| **Aspects** | **Metrics** | **Data Resolution** | **Source** |
| --- | --- | --- | --- |
| Air pollution | PM1 | 1 km | (Wei et al. 2019) |
|  | PM2.5 | 1 km | (Zhao et al. 2021) |
|  | PM10 | 1 km | (Wei et al. 2021) |
| Socioeconomic variables | Income segregation | County level | Annual Yearbook |
|  | Population density | County level | Annual Yearbook |
|  | Gross Domestic Product (GDP) | County level | Annual Yearbook |
| Urban structure | Urban Centrality Index (UCI) | County level | GAODE Web API platform |
|  | Point Of Interest (POI) density | County level | GAODE Web API platform |
|  | Road density | County level | OpenStreetMap |
| Environmental Changes | Mean Annual Temperature (MAT) | 1 km | (Peng et al. 2019) |
|  | Mean Annual Precipitation (MAP) | 1 km | (Peng et al. 2019) |
|  | Normalized Difference Vegetation Index (NDVI) | 0.05° | (Li et al. 2024) |
|  | Conversion pressure index (CPI) | 1 km | (Oakleaf et al. 2024) |
|  | Mean Annual Surface Air Pressure | 0.1° | (He et al. 2020) |
|  | Mean Annual Ventilation Coefficient | 10 m | https://climatedataguide.ucar.edu/climate-data/era-interim |

**Table S2. Descriptive statistics for urban form, socioeconomic and environmental metrics.** Abbreviations: POI, points of interest; UCI, urban centrality index; GDP, gross domestic product; CPI, Conversion pressure index; MAT, mean annual temperature; MAP, mean annual precipitation; NDVI, normalized difference vegetation index; SAP: surface air pressure; VC: ventilation coefficient.

| **Metrics** | **Mean (SD)** | **Percentile** | | | | |
| --- | --- | --- | --- | --- | --- | --- |
|  |  | **min** | **25** | **50** | **75** | **max** |
| Road density  (log m/km2) | 2.32 (3.02) | 0.24 | 0.5 | 1.01 | 2.59 | 14.03 |
| POI density  (points of POI /km2) | 96.38 (255.82) | 1.44 | 3.33 | 9.59 | 42.24 | 1441.57 |
| Population density (individuals/km^2^) | 1598.72 (4228.14) | 62.87 | 125.36 | 283.28 | 922.95 | 27982.7 |
| 2020 GDP (CNY) | 5407.61 (5878.96) | 530.45 | 2058.58 | 3516.98 | 6588.59 | 37237.94 |
| Income Segregation | 0.27 (0.09) | 0.2 | 0.21 | 0.23 | 0.28 | 0.59 |
| UCI | 0.21 (0.16) | 0.02 | 0.1 | 0.14 | 0.31 | 0.7 |
| MAT (℃) | 19.99 (1.52) | 16.32 | 18.97 | 19.87 | 21.5 | 22.4 |
| MAP (mm) | 1357.52 (329.21) | 709.16 | 1137.05 | 1354.56 | 1588.45 | 2185.87 |
| NDVI | 0.63 (0.12) | 0.2 | 0.62 | 0.69 | 0.7 | 0.72 |
| CPI | 0.62 (0.08) | 0.32 | 0.58 | 0.64 | 0.68 | 0.77 |
| SAP (hPa) | 972.19 (27.48) | 911.45 | 952.12 | 966.05 | 994.79 | 1015.39 |
| VC (m²/s) | 1261.14 (871.49) | 543.35 | 691.32 | 844.60 | 1431.45 | 5202.64 |

**References**

He, J., K. Yang, W. Tang, H. Lu, J. Qin, Y. Chen, and X. Li. 2020. The first high-resolution meteorological forcing dataset for land process studies over China. Scientific Data **7**:25.

Li, H., Y. Cao, J. Xiao, Z. Yuan, Z. Hao, X. Bai, Y. Wu, and Y. Liu. 2024. A daily gap-free normalized difference vegetation index dataset from 1981 to 2023 in China. Scientific Data **11**:527.

Oakleaf, J., C. Kennedy, N. H. Wolff, D. E. Terasaki Hart, P. Ellis, D. M. Theobald, B. Fariss, K. Burkart, and J. Kiesecker. 2024. Mapping global land conversion pressure to support conservation planning. Scientific Data **11**:830.

Peng, S., Y. Ding, W. Liu, and Z. Li. 2019. 1 km monthly temperature and precipitation dataset for China from 1901 to 2017. Earth System Science Data **11**:1931-1946.

Wei, J., Z. Li, J. Guo, L. Sun, W. Huang, W. Xue, T. Fan, and M. Cribb. 2019. Satellite-derived 1-km-resolution PM1 concentrations from 2014 to 2018 across China. Environmental science technology **53**:13265-13274.

Wei, J., Z. Li, W. Xue, L. Sun, T. Fan, L. Liu, T. Su, and M. Cribb. 2021. The ChinaHighPM10 dataset: generation, validation, and spatiotemporal variations from 2015 to 2019 across China. Environment International **146**:106290.

Zhao, F., L. Xiong, C. Wang, H. Wei, J. Ma, and G. Tang. 2021. Clustering stream profiles to understand the geomorphological features and evolution of the Yangtze River by using DEMs. Journal of Geographical Sciences **31**:1555-1574.
